# How does goal orientation affect employees’ innovation behavior: Data from China

**DOI:** 10.3389/fpsyg.2022.890062

**Published:** 2022-09-15

**Authors:** Meirong Zhen, Jinru Cao, Mi Wang

**Affiliations:** School of Management, Jiangsu University, Zhenjiang, China

**Keywords:** learning goal orientation, proving goal orientation, avoiding goal orientation, innovation behavior, psychological capital, organizational innovation climate

## Abstract

The study takes an interaction perspective to examine possible interaction effects of goal orientation, psychological capital, and organizational innovation climate aimed at enhancing employees’ innovation behavior. A total sample of 398 employees were selected in Chinese enterprises. The descriptive statistical analyses, multiple regression, and bootstrap approach are adopted to test the interactive effects after controlling for gender, age, years for work of employees, type of enterprises, and industry. Results indicate that learning goal orientation and proving goal orientation have a positive effect on employees’ innovation behavior through psychological capital. The positive relationship between psychological capital and employees’ innovation behavior is stronger when employees perceive more organizational innovation climate. Additionally, the positive effect of learning goal orientation and proving goal orientation on employees’ innovation behavior is stronger in high organizational innovation climate through high-level psychological capital than in low organizational innovation climate. However, the negative effect of avoiding goal orientation on innovation behavior is not significant. Finally, implications and further research are discussed.

## Introduction

Employees’ innovation behavior, such as producing, adopting, and practicing new ideas for work methods, processes, and products (Amabile et al., 1996), is the core behavior needed by an organization to succeed in a changing business environment (Fan et al., 2016). A growing body of literature has devoted attention to the critical role of individual and organizational factors that could potentially influence innovation behavior (Scott and Bruce, 1994; Hunter et al., 2005; Ren and Zhang, 2015; Song et al., 2020). Anderson et al. (2014) identified four main factors in different levels: individual, team, organizational, and multi-levels. Many studies have considered goal orientation, ethical leadership (Chen and Hou, 2016; Li et al., 2020), and personal trait (Lomberg et al., 2017) as individual level factors, team composition and team climate (Somech and Drach-Zahavy, 2013) as team factors, and organizational innovation climate (Hsu and Chen, 2017) and organizational

supportive climate as organizational factors. An increasing number of researchers have conducted multilevel factor studies and found that employees’ innovation behavior is influenced by individual-, organizational- or team-level factors (Kumar et al., 2022).

This study that adopted social cognitive theory analyzes the theoretical conceptual framework from the multilevel perspective. Social cognitive theory suggests a model of behavior (B), cognition and other personal (P), and environmental (E) factors as interactive determinants that influence one another bidirectionally (Bandura, 1986, 2001). According to social cognitive theory, goal orientation is an important individual cognitive factor that gives directions to behavior. Goal orientation refers to individual differences in goal preferences within achievement settings and depends on personal values, needs, and beliefs (Dweck, 1991). Goal orientation has been categorized into learning, proving, and avoiding goal orientations (VandeWalle, 1997). Individuals with different goal orientations have their respective perceptual-cognitive frameworks (Janssen and Van Yperen, 2004), thereby explaining why some employees are more innovative than others. Direct affection of goal orientation on individual innovative behavior and moderating role of goal orientation concerning innovative behavior have been considerably proven (Hirst et al., 2009; Guo et al., 2019; Ma et al., 2021). However, existing literature has not sufficiently discussed the indirect effects of goal orientation on employees’ innovation behavior. Hence, the mechanism between goal orientation and employees’ innovation behavior remains unclear and should not be disregarded.

Bandura (2001) suggested that positive psychological state is an important mechanism, resulting in the desirable outcomes, such as employee’s innovative behavior. Luthans et al. (2007) used positive psychology ideas to develop the concept and construct of the psychological capital. Psychological capital is defined as an individual’s positive psychological state of development and consisted of hope, self-efficacy, optimism, and resilience, which are crucial in the relationship between goal orientation and innovation behavior. Social cognitive theory has devoted particular attention to environmental factors on human functioning. Existing studies have suggested that compatibility between person and environment is a critical condition for enhanced creativity (Choi, 2004). Organizational innovation climate can create favorable environment for individual innovation behavior (Andersson et al., 2020; Battistelli et al., 2021). Yan et al. (2020) indicated that organizational innovation climate can significantly influence psychological capital and innovation behavior.

However, only limited studies have been conducted on the affecting mechanism of goal orientation as personal cognitive with psychological capital and innovation climate that aims for more innovation. Accordingly, the following questions should be answered: How would different categories of goal orientation affect the innovation behavior of employees with different levels of psychological capital? Is organizational innovation climate a significant condition interacting with goal orientation and psychological capital on innovation behavior? Given that these questions remained unanswered, we found theoretical importance to investigate the interaction effect of goal orientation, psychological capital, and organizational innovation climate on innovation behavior.

The objective of our research was to explore how goal orientation affects employees’ innovation behavior by taking psychological capital as a mediator and organizational innovation climate as a moderator. We contribute to the literature by explaining the relationship among goal orientation as an individual cognitive framework, psychological capital as a positive psychological state, and innovation climate as an environmental factor and innovation behavior. Employees with different goal orientations may have different levels of psychological capital and present different innovative output in response to their perceived innovation climate. The remainder of the paper is arranged as follows. The next section contains a review of the literature, which leads to the development of the hypotheses. The following section discusses the methodology used and evaluates the results of the hypotheses. Finally, theoretical and practical implications are discussed in the conclusion.

## Theoretical framework and hypotheses

### Goal orientation and innovation behavior

In the 1980s, Dweck defined goal orientation according to the research conducted with grade school children. Goal orientation refers to the individual differences in goal preferences within achievement settings. Dweck (1991) identified learning goal orientation and performance goal orientation as two major classes of goal orientations. Learning and performance goal orientations relate to different implicit theories on personal abilities as well as different beliefs on the value of effort, causes of success, and interpretation of feedback.

Originally, goal orientation was central to educational psychology literature, but it was also discussed in organizational studies (Shamin et al., 2017). VandeWalle (1997) studied goal orientation with MBA students and noted that performance goal orientation encompasses both the desire to gain favorable judgments and to avoid unfavorable judgments about one’s ability. VandeWalle (1997) claimed that goal orientation should have three dimensions, which are redefined as follows. First, learning goal orientation is a focus on developing one’s competence by acquiring new skills, mastering new

situations, and learning from experience. Second, proving goal orientation is a focus on demonstrating one’s competence and on gaining favorable judgments from others. Third, avoiding goal orientation is a focus on avoiding negation of one’s competence and negative judgments from others. These categories of goal orientation have been well established in the literature and have received substantial empirical support (Elliot and Church, 1997; VandeWalle, 1997; Lin et al., 2022). The different types of goal orientation are neither mutually exclusive nor contradictory (Button et al., 1996). They are independent of each other. Goal orientation plays a significant role in numerous organizational decisions (Shamin et al., 2017).

To distinguish the effect of learning goal orientation, proving goal orientation, and avoiding goal orientation on innovation behavior, we apply the perspectives of behavioral activation system (BAS) and behavioral inhibition system (BIS) (Sherf et al., 2021), which group human behaviors into two categories based on self-regulatory systems of goal choice (Wrosch et al., 2003). BAS is associated with obtaining potential opportunities and rewards. Individuals with BAS would be motivated by desirable goals and are future-focused. By contrast, BIS is related to avoiding potential punishments and minimizing threats (Sherf et al., 2021). Individuals with BIS prefer to avoid potential failures and prevent new negative change.

Individuals with learning goal orientations have an incremental implicit theory and perceive ability as a malleable trait that can be developed with effort and persistence (Dweck, 1991). We argue that learning goal orientations response to BAS regulations with enthusiasm and approach-oriented behaviors. For learning goal-oriented individuals, the effort is an important determinant of success, an effective path to activate one’s current ability, and a strategy to develop additional capabilities needed for future task mastery. Individuals with high levels of learning goal orientation are self-initiated, which align with BAS and are apt to achieve desirable outcomes by diagnosing progress, correcting errors, or formulating alternative strategies (Kakkar et al., 2016). Goal orientation plays a key role in creativity, and learning goal-oriented individuals would positively seek new skills and information to achieve innovation performance (Zia, 2020; Zhou, 2021). Individuals with high learning orientation tend to accept the challenges and difficulties when innovating (Annosi et al., 2020) acting as BAS, thereby possibly triggering innovation behavior continually (Sherf et al., 2021). Thus, we propose the following hypothesis:

H1a: Learning goal orientation has a positive effect on employees’ innovation behavior.

Empirical findings on proving goal orientation were more complex because several studies found that it has no significant (Janssen and Van Yperen, 2004; Song et al., 2015) or negative relationship (Zhu et al., 2019) with employee’s creativity. However, numerous studies have shown that proving goal orientation has positive associations to achievement, persistence, and innovation (Elliot and Church, 1997; Gong et al., 2013; Liu et al., 2015; Janke et al., 2016). Individuals with proving goal orientation are interested in showing their skills and competency (Khattak et al., 2017; Domurath et al., 2020). They demonstrate their competence through knowledge manipulation (Lu et al., 2012). They may promote their knowledge as a part of their persistent

endeavors and manipulate the value and content of their knowledge to maximize their performance (Rhee and Choi, 2017; Shariq et al., 2019). Li and Tsai (2020) found that employees who have proving goal orientation would perform well and improve their learning outcomes. Ma et al. (2021) indicated that proving goal orientation can strengthen their challenge behavior. We are convinced that proving goal orientation acts as BAS. By being valued as “innovative employees,” proving goal-oriented employees’ BAS would be triggered significantly and employees could receive positive judgment and enhance their confidence and ability by engaging creatively with problems and challenges (Hirst et al., 2009; Liang et al., 2019). Hence, we propose the following hypothesis:

H1b: Proving goal orientation has a positive effect on employees’ innovation behavior.

By contrast, individuals with strong avoiding goal orientation and entity implicit theory consider ability an innate and fixed attribute that is difficult to develop. Avoiding goal orientation acts as BIS, which corresponds to avoiding dangers and failures (Sherf et al., 2021). Individuals with high level of avoiding goal orientation can view substantial effort as evidence of ineffectiveness, disadvantage, and failures after hard work as an unbearable confirmation of low ability. Thus, when creativity-relevant skills are involved, avoiding goal-oriented employees have the tendency to avoid challenges or uncertainties that pose risks of error (VandeWalle, 1997; Guo et al., 2019; Shariq et al., 2019). They would not explore situations or generate innovative ideas with high possibilities of receiving negative feedback (Gong et al., 2013). Therefore, the following hypothesis is generated:

H1c: Avoiding goal orientation has a negative effect on employees’ innovation behavior.

### Psychological capital as a mediator

Psychological capital represents individual motivational propensities that accrue through positive psychological constructs, such as efficacy, optimism, hope, and resilience (Luthans and Youssef, 2004), thereby indicating one’s “positive appraisal of circumstances and probability for success based on motivated effort and perseverance” (Luthans et al., 2007, p. 550). Several studies have provided strong support that psychological capital has a positive effect on employees’ growing performance and innovation (Park and Jo, 2018; Slåtten et al., 2019; Montani et al., 2020; Yan et al., 2020). Psychological capital provides positive psychological resources that can release anxiety, uncertainty, and distress from the innovation process (Broad and Luthans, 2020). Employees with strong efficacy would like to carry out a broad and proactive set of work tasks that extend beyond prescribed technical requirements (Parker, 1998; Sun et al., 2016). Given challenging tasks, optimistic and hopeful employees generally believe that good rather than bad things will happen to them. Even though things go bad, employees with high resilience would bounce back from adversity, uncertainty, conflict, and failure to even positively carry out new ideas (Luthans, 2002). Thus, we propose the following hypothesis:

H2: Psychological capital has a positive effect on employees’ innovation behavior.

Goal orientation and psychological capital are individual factors that affect employees’ innovation behavior. Accordingly, the following question must be answered: What is the relationship among goal orientation, psychological capital, and employees’ innovation behavior? Renn and Vandenberg (1995) generally supported the mediating role of critical psychological states (CPS). Olaniyan and Hystad (2016) showed the indirect effect of authentic leadership through psychological capital on employees’ job satisfaction, insecurity, and intentions to quit. These aspects may provide some support for the mediating role of psychological capital between goal orientation and innovation behavior.

Individuals with learning goal orientation pursue an adaptive response pattern and view challenging tasks as an opportunity for growth and development. Learning goal-oriented individuals present BAS associated with positive emotional states and drive others to achieve goals through effective learning (Merchan-Clavellino et al., 2019). Learning goal orientation has been found to characterize individuals who have a generally positive approach to life, challenges, and success (Elliot and McGregor, 2001). Individuals with learning goal orientation have a positive relationship with self-efficacy (Geitz et al., 2016; Zhang et al., 2019; Du et al., 2020). By believing that ability can be developed, these individuals are receptive to finding ways to develop the skills needed to overcome setbacks (Annosi et al., 2020). Additionally, learning goal orientation has a positive relationship with optimism and hopes that foster resiliency to setbacks (Nuutila et al., 2020). Thus, individuals with learning goal orientation may exhibit high-level psychological capital, and learning goal orientation may affect innovation behavior through psychological capital. Therefore, the following hypothesis is generated:

H3a: Psychological capital mediates the relationship between learning goal orientation and employees’ innovation behavior.

Individuals with proving goal orientation act BAS and want to receive positive comments from others. They would have high levels of hope, self-efficacy and optimism, and enthusiastic to prove their abilities by demonstrating their innovation behavior (Farmer et al., 2003). This situation proves that proving goal-oriented individuals have greater confidence and competence and easy to bounce back by favorable judgment of being innovative. Thus, individuals with proving goal orientation may exhibit high-level psychological capital, and proving goal orientation may affect innovation behavior through psychological capital. Therefore, the following hypothesis is generated:

H3b: Psychological capital mediates the relationship between proving goal orientation and innovation behavior.

For avoiding goal orientation, there is a risk of failure that would demonstrate their inadequate abilities and receive negative comments from others. Individuals with avoiding goal orientation present BIS that connects to negative emotions, such as worry and fear (Sherf et al., 2021). Avoiding goal-oriented employees exhibit low self-esteem, low self-efficacy, and decreased emotional stability (Elliot and Church, 1997) and have difficulty in overcoming setbacks. Such individuals make negative attribution about abilities and report decreased interest in tasks and withdraws from such tasks. Thus, individuals with avoiding goal orientation may exhibit low-level psychological capital, and avoiding goal orientation may affect innovation behavior through psychological capital. Therefore, the following hypothesis is generated:

H3c: Psychological capital mediates the relationship between avoiding goal orientation and employees’ innovation behavior.

### Organizational innovation climate as a moderator

The interactional perspective claims that the climate in which a person works either facilitates or inhibits his or her innovation behavior (Benjamin et al., 2014; Hsu and Chen, 2017; Li et al., 2020). For organizational climate, scholarly literature reveals two main perspectives of ontological issues. Several theorists have conceived organizational climate as an objective property of the organization that exists independently of the perceptions and understandings of its members. It is a combination of feelings, attitudes, values, and behaviors that characterize research life in the organization (Ekvall, 1996). Other theorists define organizational climate as a collective perception generated from the interaction among organization members (Schneider, 1975; Amabile et al., 1996). In this study, the organizational innovative climate refers to the perception of organizational members on the procedures, rules, practices, and behaviors that can promote the creation, development, and realization of new ideas (Van der Vegt et al., 2005; Zohar and Hofmann, 2012; Ehrhart et al., 2015; Andersson et al., 2020). It is the perceived psychological climate that nurtures creative or innovative behavior which is more important than objective climate for an individual’s intrinsic motivation (Amabile et al., 1996). A work climate in which the employees feel supported can enhance their performance, particularly on exploratory activities (Deci et al., 2001). When employees perceive the support for innovation from organizations, they would return the favor from organizations by doing well in innovation-role behavior (Fan et al., 2022). In an organizational innovation climate, people are allowed to handle unexpected issues proactively and to make decisions even in situations where they lack information and certainty in the workplace (Isaksen and Ekvall, 2010).

Organizational innovation climate could create positive conditions necessary for psychological capital to flourish. When employees perceive organizational innovation climate and feel supported, they are more likely to try unproven or new methods with higher level of hope to accomplish the tasks at workplaces. Moreover, organizational innovation climate will likely act as a contextual resource for individuals to immediately bounce back after setbacks. By experiencing high levels of resiliency, employees would not be in fear of reprisal or punishment owing to their mistakes in a supportive innovation climate. An organizational innovation climate may contribute to individual optimistic levels by allowing employees to attribute failures to external circumstances versus low personal knowledge, skills, and abilities (Zhang et al., 2015). When the level of employees’ psychological capital is high, they would prefer to show their innovation behavior (Ye et al., 2021). A growing recognition is that employees would have higher level of psychological capital and cause more innovation behaviors in an organizational innovation climate. Accordingly, this research formulates the following hypothesis:

H4: Organizational innovation climate moderates the relationship between psychological capital and employees’ innovation behavior.

Organizational innovation climate provides a safe context for taking on challenges, exchanging ideas (Dirks and Ferrin, 2001), and encouraging mutual learning and cooperation among organization members. Such a climate affects employees’ attitudes and behaviors. Individuals with learning goal orientation would present more BAS that has implications for promoting positive challenge appraisal in innovation supportive climate (Espedido and Searle, 2020). On the one hand, learning goal-oriented individuals who perceive the climate appear to have more positive attitude to challenges and be more closely related to creativity (Kim and Kwon, 2017). On the other hand, learning goal-oriented individuals would establish supportive relationship with colleagues who could suggest a positive effect on innovative engagement (Schaufeli and Bakker, 2004). Organizational innovation climate would result in greater innovative behavior when perceived by individuals with high levels of learning goal orientation. Hence, we propose the following hypothesis:

H5a: Organizational innovation climate strengthens the relationship between learning goal orientation and employees’ innovation behavior.

An innovation climate could minimize potential risks in the innovation process (Zhou and George, 2001). Individuals with proving goal orientation would present more BAS that has implications for less stress appraisal in an innovation supportive climate (Espedido and Searle, 2020), thereby enhancing their confidence and ability by engaging creatively with problems and challenges (Hirst et al., 2009). Therefore, when they perceive the innovation climate, they would have greater confidence, competence, and insist on demonstrating their innovation behavior (Farmer et al., 2003). Hence, we propose the following hypothesis:

H5b: Organizational innovation climate strengthens the relationship between proving goal orientation and employees’ innovation behavior.

Organizational innovation climate acts as a safeguard against infections of failure for avoiding goal-oriented individuals and they would present less with BIS. They would have less anxiety and less failure appraisal (Mckay et al., 2020). Accordingly, organizational innovation climate may decrease the negative effect of avoiding goal orientation on employees’ innovation behavior. Hence, the following hypothesis is generated:

H5c: Organizational innovation climate weakens the relationship between avoiding goal orientation and employees’ innovation behavior.

According to H3, H4, and H5, we build a moderated mediation model. Employees with learning goal orientation and proving goal orientation would have high-level psychological capital and act positively toward more innovation behavior in high-level organizational innovation climate than in low-level organizational innovation climate. Additionally, employees with avoiding goal orientation would have fewer negative actions on innovation behavior through psychological capital in high-level organizational innovation climate than in low-level organizational innovation climate. Hence, we propose the following hypotheses:

H6a: Organizational innovation climate can positively moderate the relationship between learning goal orientation and innovation behavior though psychological capital.

H6b: Organizational innovation climate can positively moderate the relationship between proving goal orientation and innovation behavior though psychological capital.

H6c: Organizational innovation climate can negatively moderate the relationship between avoiding goal orientation and innovation behavior though psychological capital.

**FIGURE 1:**
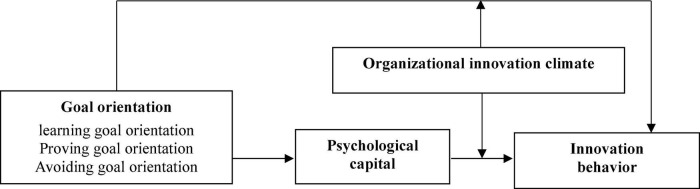
Theoretical model.

Figure 1 illustrates the theoretical model underlying the hypotheses presented in this research.

## Method

### Sample and procedure

The data for this study were collected through a survey from 600 R&D employees from 41 enterprises in China. The enterprises were randomly selected from five different provinces, namely, Jiangsu, Zhejiang, Shanxi, Henan, and Ningxia, which are located in the eastern, middle, and western areas of China. Printed questionnaires inquired about respondents’ perceptions regarding innovation behavior. For each item, those respondents described their opinions on organizational innovation climate, goal orientation, and innovation behavior. A total of 537 completed questionnaires were returned (89.5% response rate) and 398 questionnaires were valid (63.3% valid response rate). When selecting valid questionnaires, some questionnaires were excluded, such as those lacking more than five items, repeatedly selecting the same option or having contradictory answers to positive and negative questions. The sample indicated that 56.5% of the respondents were men and 43.5% were women. Among the total respondents, 43.5% of them had a bachelor’s degree, 16.8% of them had a master’s degree, and 23.4% of them had been working for over 5 years. In terms of age, respondents aged 25 and below were 21.7%, 26- to 35-year-old respondents were 30.4%, 36- to 45-year-old respondents were 31.7%, and respondents aged 46 and above were 16.2%. The enterprises were distributed among the following branches: electronic communication (15.8%), instrument manufacturing (31.0%), biomedical industry (6.2%), chemical and food industry (6.0%), computer and software service (2.8%), banking and finance (6.7%), and construction industry (31.5%). In total, 27.8% of the enterprises were state-owned enterprises, 52.6% of the enterprises were private enterprises, and 19.6% of the enterprises were international enterprises.

### Measures

The questionnaire adopted a five-point Likert scale with answers that ranged from “strongly disagree” to “strongly agree.” The original scales of innovation behavior and goal orientation were in English. They were translated into Chinese and then back into English to ensure proper understanding for respondents and simultaneously retain their original meaning.

#### Goal orientation

Goal orientation is a categorical variable with three dimensions of learning, proving, and avoiding goal orientations. These dimensions were measured using the scale of VandeWalle (2001) with 12 sub-categorized items. An example item for learning goal orientation is “I am willing to select a challenging work assignment that I can learn a lot from.” An example item for proving goal orientation is “I like to show that I can perform better than my co-workers.” An example item for avoiding goal orientation is “I would avoid taking on a new task if there was a chance that I would appear rather incompetent to others.” Cronbach’s α for the whole scale is 0.990 and for subscales is between 0.800 and 0.818, which all indicate good reliability.

#### Organizational innovation climate

The scale to measure organizational innovation climate was designed by Qiu et al. (2009). The scale was based on Work Environment Index (Amabile and Gryskiewicz, 1989) and is more suitable for Chinese. Employees assessed their perceived organizational climate in enhancing innovation and creativity. The scale has 35 items in seven dimensions, which are organizational value, operating method, resource supply, team operation, learning and growing, leadership efficiency, and organizational climate. One sample item is “My supervisor can respect and support my innovation at the workplace.” Cronbach’s α for this scale is 0.970 and for subscales is between 0.850 and 0.950, which all indicate good reliability.

#### Employees’ innovation behavior

The scale to measure and rate the level of employees’ innovation behavior was designed by Scott and Bruce (1994). In their study (Scott and Bruce, 1994), employees’ innovation behavior was rated by the supervisors. However, the study of Carmeli et al. (2006) indicated that the reliability of the same scale as reported by the employees themselves was similar to the Cronbach’s α of 0.89 reported by Scott and Bruce (1994). In this study, a self-evaluation was adapted, and innovation behavior was measured as a single-dimensional variable. The scale has six items, including “Promotes and champions ideas to others.” Cronbach’s α for this scale is 0.930, which indicates good reliability.

#### Psychological capital

The scale to measure psychological capital was designed by Luthans et al. (2007). Employees rated their positive psychological state of efficacy, hope, resilience, and optimism. The scale has 24 items in four dimensions: efficacy, hope, resilience, and optimism. One sample item is “I feel confident in representing my work area in meetings with management.” Cronbach’s α for this scale is 0.970 and for subscales is between 0.890 and 0.980, which indicate that the scale has good reliability.

#### Control variables

The effect of gender, age, years for work, type of enterprises, and industry were statistically controlled to remove their potential confounding effect.

### Confirmatory factor analysis

Confirmatory factor analysis (CFA) was conducted to assess the model fit and validity by inputting raw data to IBM SPSS AMOS and requesting an analysis based on the covariance matrix (Jöreskog and Sörbom, 1996). Fit indices indicated that six-factor model has a better fit for the hypothesized model when compared to the five-factor model, four-factor model, three-factor model, two-factor model, and one-factor model (see Table 1). All items loaded significantly on the latent constructs they were designed to measure.

**TABLE 1:** Fit indices of CFA.

CFA	χ^2^	df	χ^2^/df	RMSEA	NFI	CFI	IFI	TLI
Six-factor model	529.913	237	2.235	0.056	0.836	0.901	0.902	0.885
Five-factor model	704.812	242	2.912	0.069	0.782	0.844	0.845	0.822
Four-factor model	496.474	98	5.066	0.101	0.717	0.756	0.789	0.701
Three-factor model	430.792	101	4.265	0.090	0.754	0.798	0.800	0.760
Two-factor model	709.433	103	6.887	0.121	0.629	0.661	0.665	0.606
Single-factor model	311.648	44	7.082	0.123	0.587	0.617	0.623	0.521

Mathieu and Farr (1991) mentioned that if variables with items are more than 7, they need to be simplified into three items.

A: Learning goal orientation, B: Proving goal orientation, C: Avoiding goal orientation, D: Psychological capital, E: Organizational innovation climate, F: Innovation behavior. One-factor model: A + B + C + D + E + F; two-factor model: A + B + C + D + E, F; three-factor model: A + B + C + D, E, F; four-factor model: A + B + C, D, E, F; five-factor model: A + B, C, D, E, F; and six-factor model: A, B, C, D, E, F.

The probability of common-method variance exists because every part of the questionnaire was self-reported by employees. Thus, this study used the CFA marker technique to explore the common method variance (CMV) and unmeasured method variance (UMV) models and to conduct model comparisons with various key parameters (refer to Table 2). Key parameters that show the CMV model has greater fit than the UMV model indicate a significant common method bias (Williams et al., 2010). However, the present model comparisons show the key parameters of the CMV model that does not have greater fit than those of the UMV model, which suggests a lack of common method variance to confound the interpretation of results.

**TABLE 2:** CMV and UMV model comparisons of key parameters.

Model	χ ^2^	df	χ ^2^/df	RMSEA	AGFI	NNFI	CFI	IFI	SRMR
UMV	468.053	164	2.854	0.069	0.862	0.963	0.986	0.968	0.050
CMV	798.620	149	5.360	0.092	0.792	0.934	0.948	0.948	1.157

## Results

The direct influence of goal orientation, psychological capital, and innovation behavior and the indirect effects of mediating effect, moderating effect, and moderated mediation effect were validated by IBM SPSS AMOS software package with bootstrapping approach in this study.

### Descriptive statistics

Table 3 reports the means, standard deviations, and correlations among variables. Variance inflation factor (VIF) is the standard for testing multicollinearity. The results show that the maximum VIF value is 2.413 and is under the recommended maximum values (Hair et al., 2018), indicating that multicollinearity is not an issue in this study.

**TABLE 3:** Means, standard deviations, and correlations (*n* = 398).

Variables	Mean	SD	1.	2.	3.	4.	5.
1. Organizational innovation climate	3.623	0.677	–				
2. Learning goal orientation	3.981	0.705	0.364***	–			
3. Proving goal orientation	3.890	0.679	0.214***	0.422***	–		
4. Avoiding goal orientation	2.938	0.943	0.044	−0.155**	0.133**	–	
5. Psychological capital	3.854	0.526	0.633***	0.548***	0.374***	−0.091**	–
6. Innovation behavior	3.756	0.666	0.489***	0.531***	0.349***	−0.341**	0.677***

**p < 0.01; ***p < 0.001.

### Analysis of the influence of goal orientation, psychological capital, and innovation behavior

Table 4 shows the results of Hypotheses H1a, H1b, H1c, and H2. Learning goal orientation (β = 0.475, *p* < 0.001) and proving goal orientation (β = 0.310, *p* < 0.001) have significant positive relationship with innovation behavior. Thus, H1a and H1b are supported. There is no significant relationship between avoiding goal orientation and innovation behavior (β = 0.007, *p* > 0.05). Thus, H1c is not supported. The results indicate that learning goal orientation and proving goal orientation can positively affect employees’ innovation behavior. However, negative affection of avoiding goal orientation on innovation behavior is not significant. Table 4 indicates a significant relationship between psychological capital and innovation behavior (β = 0.626, *p* < 0.001). Thus, H2 is supported.

**TABLE 4:** Results of the influence of goal orientation, psychological capital on innovation behavior.

Path	Estimate	SE	CR	*P*
Learning goal orientation → Innovation behavior	0.475	0.070	6.781	0.000
Proving goal orientation → Innovation behavior	0.310	0.059	5.185	0.000
Avoiding goal orientation → Innovation behavior	0.007	0.029	0.239	0.812
Psychological capital → Innovation behavior	0.626	0.072	8.657	0.000

### Mediating effect of psychological capital

The results shown in Table 5 indicate that the mediating effect of psychological capital between learning goal orientation and innovation behavior is significant (β = 0.225, *p* < 0.05), and the bootstrap confidence interval (CI) with 95% confidence level is (0.146, 0.339), not including 0. Moreover, the mediating effect of psychological capital between proving goal orientation and innovation behavior is significant (β = 0.216, *p* < 0.01), and CI is (0.138, 0.345). Hence, H3a and H3b are supported. That is, through psychological capital, learning goal orientation and proving goal orientation positively affect employees’ innovation behavior. However, the mediating effect of psychological capital for avoiding goal orientation is not significant, and CI is (−0.095, 0.012), which includes 0. Therefore, H3c is not supported. That is, psychological capital does not mediate the relationship between avoiding goal orientation and innovation behavior when avoiding goal orientation could not affect innovation behavior significantly.

**TABLE 5:** Mediating effect test of psychological capital.

Mediating influence path	Estimate	SE	CR	Percentile 95% CI		
				
				Lower limit	Upper limit	*P*
Learning goal orientation → Psychological capital → Innovation behavior	0.225	0.047	4.787	0.146	0.339	0.010
Proving goal orientation → Psychological capital → Innovation behavior	0.216	0.053	4.075	0.138	0.345	0.007
Avoiding goal orientation → Psychological capital → Innovation behavior	−0.036	0.027	1.333	−0.095	0.012	0.169

### Moderating effect of organizational innovation climate

Table 6 shows the moderating effect of organizational innovation climate. The moderating effect of organizational innovation climate on the relationship of psychological capital and innovation behavior is significant (β = 0.399, *p* < 0.01), with 95% CI at (0.251, 0.583). Hence, H4 is supported. The moderating effect of organizational innovation climate on the relationship between learning goal orientation and innovation behavior is significant (β = 0.795, *p* < 0.05), with 95% CI at (0.462, 1.215). Similarly, the moderating effect of organizational innovation climate on the relationship between proving goal orientation and innovation behavior is significant (β = 0.404, *p* < 0.05), with 95% CI at (0.241, 0.588). Hence, H5a and H5b are supported. However, there is no significant moderating effect of organizational innovation climate on avoiding goal orientation and innovation behavior. Therefore, H5c is not supported.

**TABLE 6:** Moderating effect test of organizational innovation climate.

Moderating influence path	Estimate	SE	CR	Percentile 95% CI		
				
				Lower limit	Upper limit	*P*
Psychological capital → Organizational innovation climate → Innovation behavior	0.399	0.083	4.807	0.251	0.583	0.007
Learning goal orientation → Organizational innovation climate → Innovation behavior	0.795	0.187	4.251	0.462	1.215	0.014
Proving goal orientation → Organizational innovation climate → Innovation behavior	0.404	0.084	4.810	0.241	0.588	0.010
Avoiding goal orientation → Organizational innovation climate → Innovation behavior	−0.049	0.029	1.380	−0.098	0.010	0.113

### Moderated mediation effect of psychological capital and organizational innovation climate

The results of moderated mediation analysis are shown in Table 7. For learning goal orientation, the mediation effect of psychological capital is not significant when the organizational innovation climate is low and 95% CI is (−0.112, 0.291). In addition, mediation effect is significant (β = 0.374, *p* < 0.05) when organizational innovation climate is high and 95% CI is (0.136, 0.663), not including 0. Thus, when organizational innovation climate is higher, the effect of learning goal orientation on innovation behavior though psychological capital is stronger, which means that the moderated mediation effect is significant. Hence, H6a is supported. For proving goal orientation, mediation effect is significant (β = 0.321, *p* < 0.05), with 95% CI at (0.112, 0.586) in high organizational innovation climate. Thus, H6b is supported. However, for avoiding goal orientation, 95% CI includes 0, whether organizational innovation climate is low or high. Hence, H6c is not supported.

**TABLE 7:** Moderated mediation effect test of psychological capital and organizational innovation climate.

Moderated mediation influence path		Estimate	SE	CR	Percentile 95% CI		
					
					Lower limit	Upper limit	*P*
Learning goal orientation → Psychological capital → Innovation behavior	Low organizational innovation climate	0.071	0.101	0.703	−0.112	0.291	0.160
	High organizational innovation climate	0.374	0.133	2.812	0.136	0.663	0.023
Proving goal orientation → Psychological capital → Innovation behavior	Low organizational innovation climate	0.061	0.092	0.663	−0.142	0.233	0.444
	High organizational innovation climate	0.321	0.121	2.652	0.112	0.586	0.015
Avoiding goal orientation → Psychological capital → Innovation behavior	Low organizational innovation climate	0.015	0.023	0.652	−0.036	0.060	0.132
	High organizational innovation climate	0.067	0.043	1.558	−0.012	0.156	0.059

The preceding results show the moderated mediation relationships. For employees in high organizational innovation climate, the effects of learning goal orientation and proving goal orientation on innovation behavior are stronger through psychological capital compared with those in low organizational innovation climate.

## Discussion and conclusion

This study findings contribute to the relationship between goal orientation and employees’ innovation behavior by interacting with psychological capital and organizational innovation climate. The results indicate the relationship between goal orientation and innovation behavior in clear manner.

### Theoretical significance

First, this study took goal orientation as an individual cognitive factor based on social cognitive theory and verified that different categories of goal orientation have different relationship with employees’ innovation behavior. Learning goal orientation and performance goal orientation have direct positive effect on employees’ innovation behavior. These results are the same as the results of many previous studies (Gong et al., 2013; Zhou, 2021). However, contrary to our expectations, avoiding goal orientation showed no significant negative effect on innovation behavior.

Second, this study explored the mediating mechanism between goal orientation and innovation behavior. Considering psychological capital as the positive psychological state of individuals that could considerably predict innovation behavior (Bandura, 2001), employees having different goal orientation would have different levels of psychological capital and likely have different tendencies to innovation behavior. The results indicate that psychological capital functions as a catalyst and clarifies the inner mechanism of how goal orientation affects innovation behavior. That differs from many studies that have focused on the direct relationship between goal orientation and innovation behavior or have taken goal orientation as moderator or mediator (Guo et al., 2019; Zia, 2020) to predict innovation behavior.

Third, organizational innovation climate was considered in this study as an important environmental factor according to social cognitive theory and serves as a condition to trigger employees’ innovation behavior. The moderator role of organizational innovation climate on the relationship between learning goal orientation (proving goal orientation, but not avoiding goal orientation) and employees’ innovation behavior was proved in this study. Although previous research has proven that organizational innovation climate is one of the crucial predictors of employees’ innovation behavior (Taştan, 2013; Kang et al., 2016; Hsu and Chen, 2017), only a few studies have focused on how different categories of goal orientation react to innovation behavior in an innovation supportive climate. This study extends social cognitive theory with how individuals with different goal orientation (P) act toward innovation behavior (B) by interacting with organizational innovation climate (E).

Finally, to clarify the interaction between psychological capital and organizational innovation climate, this study developed a moderated mediation effect among variables as an initial attempt to show that in organizational innovation climate, how different types of goal orientation affect innovation behavior go through psychological capital. The result supports our expectation that psychological capital is stronger when compared to innovation behavior when employees have high, rather than low, perceived organizational innovation climate. In addition, learning goal orientation and proving goal orientation could bring more innovation behavior caused by high-level psychological capital in high organizational innovation climate than in low-level organizational innovation climate. However, the moderated mediation effect does not work for avoiding goal orientation.

### Practical significance

This study also presents practical implications for management. First, enterprises should pay greater attention to employees’ goal orientations. Enterprises should hire more learning goal-oriented or proving goal-oriented employees to build innovation teams. Employees with learning goal orientations can keep learning, developing skills and knowledge, and producing innovation behavior. Employees with proving goal orientation could positively involve in more innovation. However, enterprises should avoid recruiting individuals with avoiding goal orientation for innovational programs (Weidinger et al., 2016). Second, the study suggests that enterprises should improve the psychological capital level of employees. Psychological capital can be managed and developed as human resource capital, and the managing and developing of psychological capital have lower cost and more return than the managing and developing of other capitals (Luthans and Youssef, 2004). Luthans et al. (2007) and Zhao (2011) have researched on the management and development of psychological capital and provided the methods for enterprises. Third, enterprises need to provide a secure and supportive climate to encourage employees to act in unconventional ways to gain innovation value. A strong climate for innovation and psychological safety may lead to greater innovation behavior.

### Limitations and avenues for future research

To interpret the findings of this study, we must consider several limitations that may also suggest directions for future research. First, this study used a cross-sectional design, which could prohibit causal inferences and reduce the credibility of results. Thus, longitudinal or semi-laboratory studies are necessary to expound on the relationship among factors from the organizational climate level, factors from the individual level, and innovation behavior in future studies. Furthermore, individual-level variables, such as individuals’ risk attitude and dispositional optimism, and organizational-level variables, such as the company’s incentive structure, are ignored. These variables may be relevant to the attitude toward innovation in employees’ and company’s perspectives. In the future research, individuals’ risk attitude, dispositional optimism, and company’s incentive structure should be considered as control variables.

## Data availability statement

The raw data supporting the conclusions of this article will be made available by the authors, without undue reservation.

## Author contributions

All authors contributed to the article and approved the submitted version.

## References

[B1] Amabile T. M. Conti R. Coon H. Lazenby J. Herron M. (1996). Assessing the work environment for creativity. *Acad. Manag. J.* 39 1154–1184. 10.5465/256995 256995

[B2] Amabile T. M. Gryskiewicz N. (1989). The creative environment scales: The work environment inventory. *Creat. Res. J.* 2 231–254. 10.1080/10400418909534321

[B3] Anderson N. Potoènik K. Zhou J. (2014). Innovation and creativity in organizations: A state-of-the-science review, prospective commentary, and guiding framework. *J. Manag.* 40 1297–1333. 0149206314527128 10.1177/0149206314527128

[B4] Andersson M. Moen O. Brett P. O. (2020). The organizational climate for psychological safety: Associations with SMEs’ innovation capabilities and innovation performance. *J. Eng. Technol. Manag.* 55:101554. 10.1016/j.jengtecman

[B5] Annosi M. C. Monti A. Martini A. (2020). Individual learning goal orientations in self-managed team-based organizations: A study on individual and contextual variables. *Creat. Innov. Manag.* 29 528–545. 10.1111/caim.12377

[B6] Bandura A. (1986). *Social Foundations of Thought and Action: A Social Cognitive Theory.* Hoboken: Prentice-Hall.

[B7] Bandura A. (2001). Social cognitive theory: An agentic perspective. *Annu. Rev. Psychol.* 52 1–26. 10.1146/annurev.psych.52.1.1 11148297

[B8] Battistelli A. Odoardi C. Cangialosi N. Di Napoli G. Piccione L. (2021). The role of image expectations in linking organizational climate and innovative work behaviour. *Eur. J. Innov. Manag.* 25 204–222. 10.1108/EJIM-01-2021-0044

[B9] Benjamin R. H. Rita B. Felix B. C. (2014). Does an adequate team climate for learning predict team effectiveness and innovation potential? A psychometric validation of the team climate questionnaire for learning in an organizational context. *Procedia Soc. Behav. Sci.* 114 543–550. 10.1016/j.sbspro.2013.12.744

[B10] Broad J. D. Luthans F. (2020). Positive resources for psychiatry in the fourth industrial revolution: Building patient and family focused psychological capital (PsyCap). *Int. Rev. Psychol.* 32 542–554. 10.1080/09540261 33284046

[B11] Button S. B. Mathieu J. E. Zajac D. M. (1996). Goal orientation in organizational research: A conceptual and empirical foundation. *Organ. Behav. Hum. Decis. Process.* 67 26–48. 10.1006/obhd.1996.0063

[B12] Carmeli A. Meitar R. Wdisberg J. (2006). Self-leadership skills and innovative behavior at work. *Int. J. Manpow.* 27 75–90. 10.1108/01437720610652853

[B13] Chen A. S. Y. Hou Y. H. (2016). The effects of ethical leadership, voice behavior and climates for innovation on creativity: A moderated mediation. *Leadersh. Q.* 27 1–13. 10.1016/j.leaqua.2015.10.007

[B14] Choi J. N. (2004). Person-environment fit and creative behavior: Differential impacts of supplies-values and demands-abilities versions of fit. *Hum. Relat.* 57 531–552. 10.1177/0018726704044308

[B15] Deci E. L. Ryan R. M. Gagneì M. Leone D. R. Usunov J. Kornazheva B. P. (2001). Need satisfaction, motivation, and well-being in the work organizations of a former eastern bloc country: A cross-cultural study of self-determination. *Pers. Soc. Psychol. Bull.* 27 930–942. 10.1177/0146167201278002

[B16] Dirks K. T. Ferrin D. L. (2001). The role of trust in organizational settings. *Organ. Sci.* 12 450–467. 10.1287/orsc.12.4.450.10640 19642375

[B17] Domurath A. Coviello N. Patzelt H. Ganal B. (2020). New venture adaptation in international markets: A goal orientation theory perspective. *J. World Bus.* 55:101019. 10.1016/j.jwb.2019.101019

[B18] Du K. Wang Y. Ma X. Lou Z. Wang L. Shi B. (2020). Achievement goals and creativity: The mediating role of creative self-efficacy. *Educ. Psychol.* 40 1249–1269. 01443410.2020.18 06210 10.1080/01443410.2020.1806210

[B19] Dweck C. S. (1991). *Self-Theories and Goals: Their Role in Motivation, Personality and Development.* Nebraska: University of Nebraska Press. 2130257

[B20] Ehrhart M. G. Schneider B. Macey W. H. (2015). Organizational climate and culture: An introduction to theory, research and practice. *Pers. Psychol.* 68 703–706. 10.1111/peps.12113_3

[B21] Ekvall G. (1996). Organizational climate for creativity and innovation. *Eur. J. Work Organ. Psychol.* 5 105–123. 10.1080/13594329608414845

[B22] Elliot A. J. Church M. A. (1997). A hierarchical model of approach and avoidance achievement motivation. *J. Pers. Soc. Psychol.* 72 218–232. 10.1037/0022-3514.72.1.218 10234849

[B23] Elliot A. J. McGregor H. A. (2001). A 2\times 2 achievement goal framework. *J. Pers. Soc. Psychol.* 80 501–519. 11300582 10.1037/0022-3514.80.3.501

[B24] Espedido A. Searle B. J. (2020). Daily proactive problem-solving and next day stress appraisals: The moderating role of behavioral activation. *Anxiety Stress Coping* 33 416–428. 10.1080/10615806.2020 32290686

[B25] Fan C. Tang S. Chen L. Sun T. (2022). Perceived organizational support and proactive innovation behavior: The mediating role of basic psychological needs. *Front. Psychol.* 13:804363. 10.3389/fpsyg.2022 PMC8971370 35369198

[B26] Fan H. L. Chang P. F. Albanese D. Wu J. J. Yu M. J. Chuang H. J. (2016). Multilevel influences of transactive memory systems on individual innovative behavior and team innovation. *Think Skills Creat.* 19 49–59. 10.1016/j.tsc.2015.11.001

[B27] Farmer S. M. Tierney P. Kung-Mcintyre K. (2003). Employee creativity in Taiwan: An application of role identity theory. *Acad. Manag. J.* 46 618–630. 10.5465/30040653 30040653

[B28] Geitz G. Joosten-ten Brinke D. Kirschner P. A. (2016). Changing learning behaviour: Self-efficacy and goal orientation in PBL groups in higher education. *Int. J. Educ. Res.* 75 146–158. 10.1016/j.ijer.2015.11.001

[B29] Gong Y. Kim T. Lee D. Zhu J. (2013). A multilevel model of team goal orientation, information exchange, and creativity. *Acad. Manag. J.* 56 827–851. 10.5465/amj.2011.0177

[B30] Guo Y. Wang C. Feng Y. (2019). The impact of psychological climate on employees’ innovative use of information systems: The moderating role of goal orientation. *Behav. Inf. Technol.* 38 345–360. 10.1080/0144929X.2018.1534988

[B31] Hair J. F. Black W. C. Babin B. J. Anderson R. E. (2018). *Multivariate Data Analysis Eighth Edition.* Andover: Cengage.

[B32] Hirst G. Van K. D. Zhou J. (2009). A cross-level perspective on employee creativity: Goal orientation, team learning behavior, and individual creativity. *Acad. Manag. J.* 52 280–293. 10.5465/amj.2009.37308035

[B33] Hsu M. A. Chen F. H. (2017). The cross-level mediating effect of psychological capital on the organizational innovation climate-employee innovative behavior relationship. *J. Creat. Behav.* 51 128–139. 10.1002/jocb.90

[B34] Hunter S. T. Bedell K. E. Mumford M. D. (2005). Dimension of creative climate: A general taxonomy. *Int. J. Creat. Problem Solving* 15 97–116.

[B35] Isaksen S. G. Ekvall G. (2010). Managing for innovation: The two faces of tension in creative climates. *Creat. Innov. Manag.* 19 73–88. 10.1111/j.1467-8691.2010.00558.x

[B36] Janke S. Nitsche S. Praetorius A. K. Benning K. Fasching M. Dresel M. (2016). Deconstructing performance goal orientations: The merit of a dimensional approach. *Learn. Individual Differ.* 50 113–146. j.lindif.2016.08.013 10.1016/j.lindif.2016.08.013

[B37] Janssen O. Van Yperen N. W. (2004). Employee’s goal orientations, the quality of leader member exchange, and the outcomes of job performance and job satisfaction. *Acad. Manag. J.* 47 368–384. 20159587 10.5465/20159587

[B38] Jöreskog K. G. Sörbom D. (1996). *LISREL 8: User’s Reference Guide.* Lincolnwood: Scientific Software International.

[B39] Kakkar H. Tangiralas S. Srivastava N. K. Kamdar D. (2016). The dispositional antecedents of promotive and prohibitive voice. *J. Appl. Psychol.* 101 1342–1351. 10.1037/apl0000130 27599091

[B40] Kang J. H. Matusik J. G. Kim T. Y. Phillips J. M. (2016). Interactive effects of multiple organizational climates on employee innovative behavior in entrepreneurial firms: A cross level investigation. *J. Bus. Venture.* 31 628–642. 10.1016/j.jbusvent.2016.08.002

[B41] Khattak S. R. Saleem Z. Khan H. (2017). Relationship between goal orientation and employee creativity: A mediating role of creative self-efficacy. *Int. J. Organ. Leadersh.* 6 434–443. 10.33844/IJOL.2017.60338

[B42] Kim B. N. Kwon S. M. (2017). The link between hypomania risk and creativity: The role of heightened behavioral activation system (BAS) sensitivity. *J. Affect. Disord.* 215 9–14. 10.1016/j.jad.2017.02.033 28288308

[B43] Kumar D. Upadhyay Y. Yadav R. Goyal A. K. (2022). Psychological capital and innovative work behaviour: The role of mastery orientation and creative self-efficacy. *Int. J. Hosp. Manag.* 102:103157. 10.1016/j.ijhm.2022.103157

[B44] Li C. Makhdoom H. U. R. Asim S. (2020). Impact of entrepreneurial leadership on innovative work behavior: Examining mediation and moderation mechanisms. *Psychol. Res. Behav. Manag.* 13 105–118. 10.2147/PRBM.S236876 32099488 PMC6996225

[B45] Li D. C. Tsai C. Y. (2020). Antecedents of employees’ goal orientation and the effects of goal orientation on e-learning outcomes: The roles of intra-organizational environment. *Sustainability* 12:4759. 10.3390/su12114759

[B46] Liang H. Sun W. Fonseka M. M. Zhou F. (2019). Goal orientations, absorptive capacity, and NPD team performance: Evidence from China. *Chin. Manag. Stud.* 13 489–510. 10.1108/CMS-01-2018-0389

[B47] Lin Y. Yang M. Quade M. J. Chen W. (2022). Is the bottom line reached? An exploration of supervisor bottom-line mentality, team performance avoidance goal orientation and team performance. *Hum. Relat.* 75 349–372. 10.1177/00187267211002917

[B48] Liu D. Wang S. Wayne S. J. (2015). Is being a good learner enough? An examination of the interplay between learning goal orientation and impression management tactics on creativity. *Pers. Psychol.* 68 109–142. 10.1111/peps.12064

[B49] Lomberg C. Kollmann T. Stockmann C. (2017). Different styles for different needs-the effect of cognitive styles on idea generation. *Creat. Innov. Manag.* 26 49–59. 10.1111/caim.12188

[B50] Lu L. Lin X. Leung K. (2012). Goal orientation and innovative performance: The mediating roles of knowledge sharing and perceived autonomy. *J. Appl. Soc. Psychol.* 42 180–197. 10.1111/j.1559-1816.2012.01018.x

[B51] Luthans F. (2002). The need for and meaning of positive organizational behavior. *J. Organ. Behav.* 23 695–706. 10.1002/job.165

[B52] Luthans F. Avolio B. J. Avey J. B. Norman S. M. (2007). Positive psychological capital: Measurement and relationship with performance and satisfaction. *Pers. Psychol.* 60 541–572. 10.1111/j.1744-6570.2007.00083.x

[B53] Luthans F. Youssef C. M. (2004). Human, social and now positive psychological capital management: Investing in people for competitive advantage. *Organ. Dyn.* 33 143–160. 10.1016/j.orgdyn.2004

[B54] Ma J. Peng Y. Wu B. (2021). Challenging or hindering? The roles of goal orientation and cognitive appraisal in stressor-performance relationships. *J. Organ. Behav.* 42 388–406. 10.1002/job.2503

[B55] Mathieu J. E. Farr J. L. (1991). Further evidence for the discriminant validity of measures of organizational commitment, job involvement, and job satisfaction. *J. Appl. Psychol.* 76 127–133. 10.1037/0021-9010.76.1.127

[B56] Mckay D. Yang H. Elhai J. Asmundson G. (2020). Anxiety regarding contracting COVID-19 related to interoceptive anxiety sensations: The moderating role of disgust propensity and sensitivity. *J. Anxiety Disord.* 73:102233. 10.1016/j.janxdis.2020.102233 32442880 PMC7194061

[B57] Merchan-Clavellino A. Alameda-Bailen J. R. García A. Z. Guil R. (2019). Mediating effect of trait emotional intelligence between the behavioral activation system (BAS)/behavioral inhibition system (BIS) and positive and negative affect. *Front. Psychol.* 10:424. 10.3389/fpsyg.2019.00424 30890980 PMC6411706

[B58] Montani F. Vandenberghe C. Khedhaouria A. Courcy F. (2020). Examining the inverted u-shaped relationship between workload and innovative work behavior: The role of work engagement and mindfulness. *Hum. Relat.* 73 59–93. 10.1177/0018726718819055

[B59] Nuutila K. Tapola A. Tuominen H. Kupiainen S. Pásztor A. Niemivirta M. (2020). Reciprocal predictions between interest, self-efficacy, and performance during a task. *Front. Educ.* 5:36. 10.3389/feduc.2020.00036

[B60] Olaniyan O. S. Hystad S. W. (2016). Employees’ psychological capital, job satisfaction, insecurity, and intentions to quit: The direct and indirect effects of authentic leadership. *J. Work Organ. Psychol.* 32 163–171. 10.1016/j.rpto.2016.09.003

[B61] Park S. Jo J. S. (2018). The impact of proactivity, leader-member exchange, and climate for innovation on innovative behavior in the Korean government sector. *Leadersh. Organ. Dev. J.* 39 130–149. 10.1108/LODJ-09-2016-0216

[B62] Parker S. K. (1998). Enhancing role breadth self-efficacy: The roles of job enrichment and other organizational interventions. *J. Appl. Psychol.* 83 835–852. 10.1037//0021-9010.83.6.835 9885197

[B63] Qiu H. Chen Y. Lin B. (2009). Development and inspection of organizational innovation climate scale. *Method J.* 56 69–97. 10.1016/j.envint.2021.106629 34144478

[B64] Ren F. Zhang J. (2015). Job stressors, organizational innovation climate, and employees’ innovative behavior. *Creat. Res. J.* 27 16–23. 10.1080/10400419.2015.992659

[B65] Renn R. W. Vandenberg R. J. (1995). The critical psychological states: An underrepresented component in job characteristics model research. *J. Manag.* 21 279–303. 10.1016/0149-2063(95)90059-4

[B66] Rhee Y. W. Choi J. N. (2017). Knowledge management behavior and individual creativity: Goal orientations as antecedents and in-group social status as moderating contingency. *J. Organ. Behav.* 38 813–832. 10.1002/job.2168

[B67] Schaufeli W. B. Bakker A. B. (2004). Job demands, job resources, and their relationship with burnout and engagement: A multi-sample study. *J. Organ. Behav.* 25 293–315. 10.1002/job.248

[B68] Schneider B. (1975). Organizational climate: An essay. *Pers. Psychol.* 28 361–388. 10.1111/j.1744-6570.1975.tb01386.x

[B69] Scott S. G. Bruce R. A. (1994). Determinants of innovative behavior: A path model of individual innovation in the workplace. *Acad. Manag. J.* 37 580–607. 10.5465/256701 256701

[B70] Shamin S. Cang S. Yu H. (2017). Supervisory orientation, employee goal orientation, and knowledge management among front line hotel employees. *Int. J. Hosp. Manag.* 62 21–32. 10.1016/j.ijhm.2016.11.013

[B71] Shariq S. M. Mukhtar U. Anwar S. (2019). Mediating and moderating impact of goal orientation and emotional intelligence on the relationship of knowledge oriented leadership and knowledge sharing. *J. Knowl. Manag.* 23 332–350. 10.1108/JKM-01-2018-0033

[B72] Sherf E. N. Parke M. R. Isaakyan S. (2021). Distinguish voice and silence at work: Unique relationship with perceived impact, psychological safety, and burnout. *Acad. Manag. J.* 64 114–148. 10.5465/amj.2018.1428

[B73] Slåtten T. Lien G. Horn C. M. F. Pedersen E. (2019). The links between psychological capital, social capital, and work-related performance-a study of service sales representatives. *Total Qual. Manag. Bus. Excell.* 30 S195–S209. 10.1080/14783363.2019.1665845

[B74] Somech A. Drach-Zahavy A. (2013). Translating team creativity to innovation implementation: The role of team composition and climate for innovation. *J. Manag.* 39 684–708. 10.1177/0149206310394187

[B75] Song W. Yu H. Zhang Y. Jiang W. (2015). Goal orientation and employee creativity: The mediating role of creative role identity. *J. Health Organ. Manag.* 21 82–97. 10.1017/jmo.2014.64

[B76] Song Z. Gu Q. Cooke F. L. (2020). The effects of high-involvement work systems and shared leadership on team creativity: A multilevel investigation. *Hum. Res. Manag.* 59 201–213.

[B77] Sun H. Ni J. Wu H. Zhou L. (2016). An empirical research on the relationship between psychological capital and performance of knowledge workers. *Sci. Res. Manag.* 37 60–69.

[B78] Taştan S. B. (2013). The influences of participative organizational climate and self-leadership on innovative behavior and the roles of job involvement and proactive personality: A survey in the context of SMEs in Izmir. *Soc. Behav. Sci.* 75 407–419. 10.1016/j.sbspro.2013.04.045

[B79] Van der Vegt G. S. Van de Vliert E. Huang X. (2005). Location-level links between diversity and innovative climate depend on national power distance. *Acad. Manag. J.* 48 1171–1182. 10.5465/amj.2005.19573116

[B80] VandeWalle D. (1997). Development and validation of a work domain goal orientation instrument. *Educ. Psychol. Meas.* 57 995–1015. 10.1177/0013164497057006009

[B81] VandeWalle D. (2001). Goal orientation: Why wanting to look successful doesn’t always lead to success. *Organ. Dyn.* 30 162–171. 10.1016/S0090-2616(01)00050-X

[B82] Weidinger A. F. Spinath B. Steinmayr R. (2016). Why does intrinsic motivation decline follow negative feedback? The mediating role of ability self-concept and its moderation by goal orientations. *Learn. Individual Differ.* 47 117–128. 10.1016/j.lindif.2016.01.003

[B83] Williams L. J. Hartman N. Cavazotte F. (2010). Method variance and marker variables: A review and comprehensive CFA marker technique. *Organ. Res. Methods* 13 477–514. 10.1177/1094428110366036

[B84] Wrosch C. Scheier M. F. Miller G. E. Schulz R. Carver C. S. (2003). Adaptive self-regulation of unattainable goals: Goal disengagement, goal reengagement, and subjective well-being. *Pers. Soc. Psychol. Bull.* 29 1494–1508. 10.1177/0146167203256921 15018681

[B85] Yan D. Wen F. Li X. Zhang Y. (2020). The relationship between psychological capital and innovation behaviour in Chinese nurses. *J. Nurs. Manag.* 28 471–479. 10.1111/jonm.12926 31811781

[B86] Ye P. Liu L. Tan J. (2021). Creative leadership, innovation climate and innovation behavior: The moderating role of knowledge sharing in management. *Eur. J. Innov. Manag.* 25 1092–1114. 10.1108/EJIM-05-2020-0199

[B87] Zhang J. Cao C. Shen S. Qian M. (2019). Examining effects of self-efficacy on research motivation among Chinese university teachers: Moderation of leader support and mediation of goal orientations. *J. Psychol.* 153 414–435. 10.1080/00223980.2018.1564230 30668282

[B88] Zhang S. Yuan J. Li X. (2015). The impact of team culture and psychological capital on graduate students’ knowledge sharing behavior. *J. Grad. Educ.* 30 39–45.

[B89] Zhao S. (2011). Review on the theory and practical application of intellectual capital and psychological capital. *Soc. Sci. Nanjing* 2 22–29.

[B90] Zhou J. George J. M. (2001). When job dissatisfaction leads to creativity: Encouraging the expression of voice. *Acad. Manag. J.* 44 682–696. 10.5465/3069410 3069410

[B91] Zhou K. (2021). The influence of creative personality and goal orientation on innovation performance. *Front. Psychol.* 12:634951. 10.3389/fpsyg.2021.634951 33897542 PMC8062702

[B92] Zhu Y. Chen T. Wang M. Jin Y. Wang Y. (2019). Rivals or allies: How performance-prove goal orientation influences knowledge hiding. *J. Organ. Behav.* 40 849–868. 10.1002/job.2372

[B93] Zia N. U. (2020). Knowledge-oriented leadership, knowledge management behaviour and innovation performance in project-based SMEs The moderating role of goal orientations. *J. Knowl. Manag.* 24 1819–1839. 10.1108/JKM-02-2020-0127

[B94] Zohar D. M. Hofmann D. A. (2012). “Organizational culture and climate,” in *The Oxford Handbook of Organizational Psychology*, ed. Kozlowski S. W. J. (New York, NY: Oxford University Press).

